# A Virtual Integrated General Practitioner–Pediatrician Model of Care Implemented in Metropolitan and Rural Primary Care Settings: Qualitative Analysis of Clinician Perspectives on the SUSTAIN Model of Care

**DOI:** 10.2196/86707

**Published:** 2026-05-05

**Authors:** Susan Bullock, Raghu Lingam, Karen Wheeler, Tammy Meyers Morris, Corin Miller, Louisa Adams, Ken Peacock, Annemarie Christie, Michael Hodgins, Harriet Hiscock, Lena Sanci, Natalie Taylor, Carmen Crespo-Gonzalez

**Affiliations:** 1 Population Child Health Research Group, School of Clinical Medicine, UNSW Sydney, Building C29 HTH Level 3 North, 55 Botany St, Kensington, NSW, 2033, Australia, +61 293851000; 2 Sydney Children’s Hospitals Network, Sydney, Australia; 3 Department of Paediatrics, The University of Melbourne, Melbourne, Australia; 4 Health Services and Economics, Murdoch Children's Research Institute, Melbourne, Australia; 5 Faculty of Medicine, Health and Dental Sciences, The University of Melbourne, Melbourne, Australia; 6 Department of General Practice and Primary Care, The University of Melbourne, Melbourne, Australia; 7 School of Population Health, Faculty of Medicine and Health, UNSW Sydney, Sydney, Australia

**Keywords:** virtual health care, integrated care, rural health, pediatric health, primary care

## Abstract

**Background:**

General practitioners (GPs) play a pivotal role in a patient’s health care journey. However, demands on general practice, including complex patient management, workforce shortages, and health system fragmentation, have been shown to adversely impact the delivery of high-quality care and health outcomes. Integrated care models, particularly those that offer virtual care options, can support improved access to quality care and efficiency of health care delivery across metropolitan and rural areas. The SUSTAIN model of care was created to provide an accessible option for integrated care. It consists of centralized pediatricians supporting GPs in their practice through virtual coconsultations, virtual “lunch and learn” case discussions, and phone or email support. There is limited evaluation literature on integrated models of care being implemented in a primary care setting where the GP and family are face to face and the non-GP specialist is virtual. To address this gap, a comprehensive implementation evaluation of the SUSTAIN model of care was conducted.

**Objective:**

This study aimed to examine what, why, and how different factors impact the uptake of the SUSTAIN model of care from the perspectives of the SUSTAIN pediatricians and metropolitan and rural GPs in New South Wales, Australia.

**Methods:**

A qualitative study was conducted as part of a mixed methods implementation evaluation of the SUSTAIN model of care. Data were collected via recorded online focus groups and interviews with GPs, practice managers, and pediatricians at 6 and 12 months after the commencement of SUSTAIN. Data were analyzed thematically using iterative thematic analysis informed by the Consolidated Framework of Implementation Research.

**Results:**

Eighteen focus groups and 13 interviews were conducted. GPs, practice managers, and pediatricians found the SUSTAIN model acceptable, with the flexibility and practicality of the model highlighted. GPs valued the learning opportunities, collaboration, and support they gained from working alongside the pediatricians. Virtual delivery through telehealth was viewed as a positive means of receiving specialist support that would otherwise be inaccessible to many practices. Increased efficiency in workflow and working at the top of scope in pediatric care as well as opportunities for meaningful professional relationships and increased family trust in GP-delivered care were recognized as key benefits that enhanced uptake. The current landscape of Australian general practice, with fee-for-service billing and workflow pressures, was recognized as a barrier to engagement with SUSTAIN. GPs and pediatricians highlighted that more appropriate remuneration to support co-consultation is vital to the sustainability and scalability of the SUSTAIN model.

**Conclusions:**

The SUSTAIN model of care expands on our understanding of the benefits of integrated GP-pediatrician models of care in general practice by demonstrating the utility of a pediatrician supporting a GP in their practice via telehealth across metropolitan and rural environments in New South Wales, Australia.

## Introduction

Integrated care models are one way to support improved access to quality care and efficiency of health care delivery [1]. Integrated care has been defined as the structured effort to provide coordinated, person-centered, multidisciplinary care within the same or different organizations, either within health care or across the health, social, or community care sectors [2-4]. Integrated care models have demonstrated positive outcomes, including increased access for patients, interprofessional learnings and communications, health service efficiency, and cost-savings [1,5].

Integrated models of care with pediatricians coconsulting with and supporting general practitioners (GPs) in primary care have been evaluated in studies across the United Kingdom and Australia [6-8]. Results demonstrate reductions in pediatric hospital outpatient appointments, subspecialty pediatric appointments, and emergency department (ED) attendance, as well as improvements in quality of care for several common childhood conditions [6,9]. The Strengthening Care for Children (SC4C) model, based on the United Kingdom’s Children and Young People’s Health Partnership, comprised in-person GP-pediatrician coconsultations and case discussions and remote email and phone support. SC4C was delivered in 22 metropolitan general practices across Melbourne and Sydney [6-8].

However, in the Australian context, in-person integrated care models, such as SC4C, pose equity and scalability challenges especially for nonmetropolitan general practices where access to local pediatrician support is limited and GP workload capacity stretched [10]. Furthermore, 28% of Australians, or approximately 7 million people, live in rural, regional, and remote areas of the country [11]. Compared to their urban counterparts, children living in these areas experience inferior health outcomes and are more likely to be developmentally vulnerable in one or more domains in their first year of school [12,13]. Developmental vulnerabilities at school entry are strongly associated with long-term adverse outcomes, including school failure, lifelong disability, chronic diseases, mental illness, incarceration, and reduced economic opportunity, perpetuating cycles of disadvantage across generations [12,14]. These health and developmental challenges are further exacerbated by reduced access to appropriate health care services arising from geographical barriers and an increased likelihood of exposure to socioeconomic constraints [12,15,16].

The use of virtual care to support clinical practice has been highlighted as an efficient and cost-effective means for delivering and accessing quality health care services and outcomes [17,18], but some populations struggle to access this care equitably [10]. Telehealth, a virtual care option, is a broad term for health care services provided by practitioners to patients through digital communication channels that allow them to interact with patients, or other clinicians, without a physical presence [19]. The impact of COVID-19 accelerated the use of telehealth, driving more integrated ways of working across health sectors, addressing access difficulties, particularly for regional and rural areas, and overcoming health professional shortages [10,18,20,21]. Benefits arising from the use of telehealth include optimizing patient choice for health care delivery, increasing access to timely care, supporting the integration of the multidisciplinary team to enhance comprehensive care, reducing costs, increasing convenience, and continuity of care [18,22].

The SUSTAIN model of care was created in response to equity issues arising from the SC4C trial to allow for a more accessible option for pediatric integrated care in both metropolitan and nonmetropolitan general practice clinics. The SUSTAIN model consisted of centralized pediatricians consulting with and supporting GPs in their practices through virtual coconsultations and case discussions, phone or email support, and online educational opportunities.

There is limited literature on integrated models of care being implemented in a primary care setting where the GP remains the leading practitioner and consults patients face to face and non-GP specialists are in a support capacity only through telehealth. To address this gap, a comprehensive implementation evaluation of the SUSTAIN model of care was conducted alongside the outcome evaluation. Many health interventions with proven effectiveness are not consistently translated into clinical practice over time [23]. This gap highlights the need for robust evaluation of complex health interventions to understand the active ingredients driving implementation success, which is critical for supporting replication and sustainability. Without an understanding of the factors influencing the implementation of the SUSTAIN model of care among key stakeholders in different settings, its development, replication, and broader application remain limited. Identifying and understanding the contextual determinants that impact the uptake of the SUSTAIN model of care, considering the role of systemic and individual influences, in line with the Consolidated Framework for Implementation Research (CFIR), is vital to its long-term success [24]. This study aimed to identify and understand the barriers and facilitators perceived by pediatricians, GPs, and general practice staff as moderators of the implementation of the SUSTAIN model.

## Methods

### Study Design

This qualitative study was conducted as part of the mixed methods implementation evaluation of the SUSTAIN model of care, drawing on the methodology developed for the SC4C trial and detailed in the SC4C implementation evaluation protocol and the SUSTAIN trial protocol [25,26].

The CFIR and the SUSTAIN logic model [26] were used as frameworks to guide the analysis [25,27]. The interview guides for the semistructured interviews and focus groups were informed by the 5 CFIR domains (ie, the innovation, outer setting, inner setting, individuals, and implementation process) and were adapted from guides used in SC4C to be suitable for GPs practicing in both metropolitan and rural areas of New South Wales (NSW), Australia [25]. Standards recommended by the Consolidated Criteria for Reporting Qualitative Research were used to guide the reporting of results from this qualitative study [28]. A COREQ (Consolidated Criteria for Reporting Qualitative Research) checklist is included as Checklist 1.

### Intervention

The SUSTAIN model of care consisted of four components: (1) virtual GP-pediatrician coconsultations (up to 6 h/mo per general practice), (2) biweekly 1-hour virtual “lunch and learn” case discussion sessions (“lunch and learn”) with multiple practices at a time (the frequency of “lunch and learn” sessions changed after the beginning of the intervention from monthly to biweekly), and (3) same-day phone or (4) same-day email support within business hours. In addition, GPs had complimentary access to the internationally established Sydney Child Health Program (SCHP), an online modular child health training program targeted at GPs [29]. During their 12-month intervention period, participating GPs were able to engage flexibly with the model of care components based on their clinical needs, capacity, and learning style. Following this period, support was withdrawn completely, and practices moved into a sustainability period. Ongoing communication was provided in the lead-up to exiting the intervention to support transitioning out of the model. During the sustainability period, data collection was ongoing, but GPs had no further access to the SUSTAIN pediatricians or SCHP. The SUSTAIN model was delivered by 2 general pediatricians (1.0 FTE) employed by the Sydney Children’s Hospitals Network through a competitive employment process. The pediatricians were selected based on their experience as pediatric consultants, their involvement in junior physician training, and their prior work with GPs in regional and rural areas. They were prepared for the role by pediatricians who had previously served in the role for the SC4C project.

### Recruitment Procedure

An expression of interest (EOI) process was used to recruit general practice clinics, with general practice clinics across metropolitan, regional, and rural NSW being eligible to participate. An EOI was distributed by partnering primary health networks (Central and Eastern Sydney, South Western Sydney, and South Eastern NSW) for all general practice clinics in these networks or directly through the research team to other interested general practice clinics in rural, regional, and remote NSW. The EOI provided a written overview that outlined the project’s background, the eligibility criteria, and the expectations associated with participation. It also detailed the assessment and selection criteria that would be applied during the review process. General practice clinics that were interested in participating were asked to complete an application form to fill in relevant information.

Eligibility criteria for general practice clinics included location and service provision within NSW, being an accredited service or working toward accreditation, use of Best Practice or Medical Director software, and the ability to commit to the project over the designated timeline. More detailed information on the inclusion and exclusion criteria for general practice clinics is outlined in the SUSTAIN study protocol [26].

### Setting

Our sample included 19 general practice clinics located across NSW in rural and metropolitan locations. The practices were located across multiple local health districts with Modified Monash Model classifications MM1 (metropolitan areas) to MM5 (small rural towns) [30].

### Participant Selection

Purposive sampling was used to ensure a diverse sample of participants was recruited. After the SUSTAIN model of care had been running in a practice for 6 months, all participating GPs and practice managers were invited to participate in a virtual focus group. This invitation included GPs who had not engaged with any component of the model during the first 6 months, based on the information routinely collated by the research team through REDCap (Research Electronic Data Capture) and pediatrician reporting of coconsultation attendance, phone or email support access, and “lunch and learn” attendance as well as access to the SCHP. For GPs who were not actively participating in the model, the implementation evaluation team contacted them directly via email. These communications explained the importance of their involvement in a focus group or interview, particularly to elicit feedback on the reasons for their low engagement and to understand circumstances in which the SUSTAIN model may not have been perceived as useful in their practice context. This invitation to participate in a focus group or interview was also extended (via the practice manager) to any general practice clinic staff (practice nurses and administrative personnel) who were involved in the implementation of the trial. If participating GPs were unable to attend the focus group at 6 months, they were invited to participate in a one-on-one interview at a time of their convenience.

**Figure 1.**
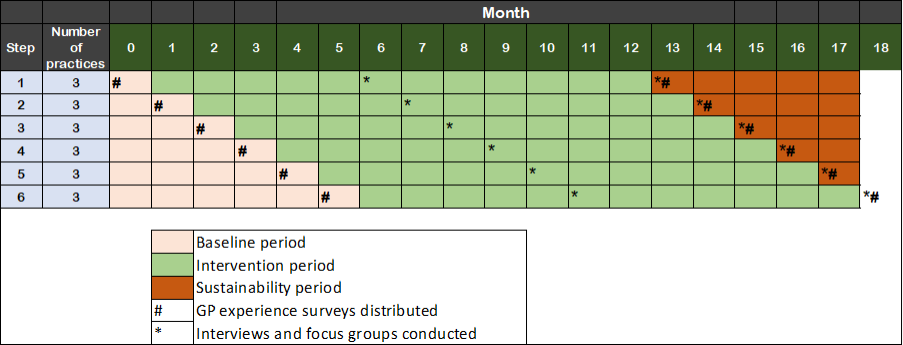
SUSTAIN proposed trial design including timing of qualitative data collection at each step. GP: general practitioner.

Following the cessation of the model of care at their practice, all participating GPs and practice managers were contacted again to participate in an end-of-intervention virtual focus group or one-on-one interview. All GPs, regardless of their level of engagement in the model, were encouraged to participate. To minimize responder bias, the SUSTAIN pediatricians did not participate in any focus groups or interviews with GPs and general practice clinic staff. After the intervention period had finalized in all practices, the SUSTAIN pediatricians were also invited to participate in either a focus group or a one-on-one interview. For this study, the term “focus group” was used to refer to any feedback session with more than 1 participant. Figure 1 provides an overview of the planned timing of the implementation evaluation of the SUSTAIN trial in the context of the broader proposed trial design.

### Data Collection

GP demographic data (eg, gender, years of practice, number of pediatric patients seen, formal pediatric training, general practice type, and billing type) were collected as part of an online GP experience survey, which was distributed to all participating GPs in the trial prior to the intervention commencing in their practice (Figure 1). Pediatrician demographic information (gender and years of practice) was collected by the research team directly. Information on the Modified Monash Location of the GP and pediatrician was collected by the research team based on practice location.

Interview and focus group data were collected between April 2024 and February 2025. All focus groups and interviews were conducted by the SUSTAIN implementation evaluation team: CCG (PhD, Research Associate, University of New South Wales [UNSW], Australia, female), SB (MClinPsych, Research Manager, UNSW, Australia, female), and CM (MBBS, PhD candidate, UNSW, Australia, female) led by CCG, an experienced implementation scientist. Where possible, focus groups were conducted by 2 interviewers. Researchers involved in the implementation evaluation came from diverse professional and personal backgrounds. To minimize the influence of prior assumptions and professional experiences on data collection and analysis, researchers approached participant engagement from a neutral standpoint. Reflexivity was embedded throughout the study through regular team discussions, critical reflection during data analysis, and ongoing consideration of how researchers’ perspectives and potential unconscious biases may shape interpretation. These reflexive practices were used to enhance the rigor, credibility, and trustworthiness of the qualitative findings.

All interviews and focus groups were completed after written informed consent was collected. Focus groups and interviews with GPs and general practice staff were facilitated virtually using a semistructured guide designed to capture experiences with and adoption of the SUSTAIN model of care. Additional discussion topics included factors moderating the implementation of the model and changes in practice resulting from participation. A modified interview guide was used for the interview with pediatricians, with discussion questions focusing on experiences of barriers and facilitators to the delivery of the intervention and recommendations for future implementation of SUSTAIN. Data collection for the qualitative interviews at 6 and 12 months continued until data saturation (ie, the point at which additional interviews no longer generated new codes, categories, or themes relevant to the research questions and codebook stabilization had been achieved). All interviews and focus groups were audio recorded through an online recording function, downloaded, and stored in a secure drive that was only accessible by the research team. All audio files were initially transcribed verbatim using the Otter.ai transcription service and downloaded and stored alongside the audio files in a secure drive. All online sources were deleted in accordance with our ethics approval. All transcripts were checked for accuracy by SB, CCG, and CM, with modifications being made if the transcript differed from the audio recording. The transcripts were deidentified in preparation for data analysis. Transcripts were not returned to participants for comment. Any words added to quotes by the researchers, to clarify meaning or deidentify locations or key details, were contained in square brackets.

### Data Analysis

Transcripts were analyzed thematically using a hybrid inductive-deductive approach using an iterative process of thematic analysis consistent with the methodological framework of Braun et al [31]. The initial phases, (1) data familiarization, (2) generation of initial codes, and (3) searching for themes, were conducted through systematic reading and verification of transcripts by CCG, SB, and CM, with line-by-line open coding independently conducted by SB and CM. The next steps, (4) grouping and reviewing themes and (5) defining and naming themes, were carried out through collaborative, in-depth discussions among CCG, SB, and CM. These discussions focused on examining the results of the line-by-line coding, including the relationships among identified themes and disagreements in the interpretation of themes. Any disagreements were resolved by jointly reexamining relevant transcript sections, considering the context around participants’ statements and drawing upon the CFIR framework to support interpretative consistency. Where necessary, additional themes or subthemes were developed by the team if these were deemed more representative of the content of the transcripts. This iterative process continued until the team reached consensus on a draft coding framework. Two additional transcripts were then further coded by SB using this draft framework, and the results of this extended coding were discussed as a team. During this discussion, any additional themes or subthemes identified by SB that lay outside the preliminary framework were critiqued collaboratively, with the relevant transcripts revisited to determine whether modifications to the coding framework were required. The same analytic approach was used at 6 and 12 months. At 6 months, saturation was reached prior to the completion of data collection. No new inductive codes emerged in the final set of interviews, indicating codebook stabilization, and subsequent data contributed primarily to the refinement and elaboration of existing themes. The 12-month interviews were analyzed against the established codebook and updated iteratively to allow for genuinely new content to emerge. The implementation team met regularly to assess whether successive interviews contributed novel codes or extended existing themes in a meaningful way. From approximately the 10th interview onward, no new inductive codes emerged; subsequent interviews corroborated and elaborated previously identified themes rather than introducing new domains. Given the observed repetition across interviews, the stability of the codebook over multiple consecutive interviews, and team consensus that additional data were unlikely to yield new insights, we judged saturation to have been reached at 12 months. The CFIR was used throughout the analysis as the guiding framework, given its capacity to offer a comprehensive and systematic approach to examining complex implementation challenges. All transcripts were uploaded to and coded in NVivo (version 12; Lumivero).

### Ethical Considerations

All participants in this study provided written consent. All data provided by participants was deidentified prior to analysis. Only members of the research team had access to participant data and ensured it was treated according to principles of privacy and confidentiality. Participating general practice clinics received a one-off AUS $1000 (US $713) payment to support implementation of the SUSTAIN trial into their practice. Participating GPs and pediatricians did not receive any additional compensation for participation in interviews or focus groups. This trial was approved by the Human Research Ethics Committee of the Sydney Children’s Hospital Network (2022/ETH02068), NSW, Australia.

## Results

### Overview

Table 1 outlines the number of GPs, practice managers, and pediatricians invited to participate at 6 and 12 months and the number of GPs, practice managers, and pediatricians who participated in either a focus group or a one-on-one interview at 6 months only, 6 and 12 months, and 12 months only. One GP was interviewed at 12 months only. All additional GPs and practice managers (n=9) who participated in the 12-month interviews or focus groups had participated in an interview or focus group at 6 months. The 2 pediatricians were only interviewed once the intervention was completed in all practices.

**Table 1.** Overview of general practitioners (GPs), practice managers, and pediatricians who were invited to and participated in focus groups and interviews and 6 and 12 mo.

	GPs			Practice managers			Pediatricians		
	6 months only	6 and 12 months	12 months only	6 months only	6 and 12 months	12 months only	6 months only	6 and 12 months	12 months only
Invited to focus group/interview, n	96	0	74^a^	18	0	17	0	0	2
Participated in focus group, n	40	3	0	5	1	0	0	0	2
Participated in one-on-one interview, n	4	5	1	1	0	0	0	0	0
Total, n	44	8	1	6	1	0	0	0	2

a Reduction in the number of GPs and practice managers invited to participate between 6 and 12 mo was due to formal withdrawal from the trial of a general practice clinic (1 practice manager) and 15 GPs between these time points.

No additional practice staff (practice nurses and administrative personnel) consented to participate in the focus groups or one-on-one interviews. Focus group duration ranged from 24 to 83 minutes. One-on-one interview duration ranged from 18 to 41 minutes. Table 2 outlines demographic information for participants of the 6- and 12-month focus groups and interviews.

An overview of the level of engagement of the participating GPs in coconsultations, phone or email support, “lunch and learn” sessions, and the SCHP is presented in Figure S1 and Table S1 in Multimedia Appendix 1.

Five key themes were identified through the thematic analysis: compatibility and adaptability of the SUSTAIN model as drivers of seamless delivery and engagement; the SUSTAIN model’s relative advantage in reinforcing GPs’ pediatric care capability, access, and motivation; GP-pediatrician relational connections and communication strengthening collaboration and trust; relative priorities, needs, and available resources as key moderators of participants’ engagement with the model; and funding and enhancement of model design: considerations for sustainability.

**Table 2.** Demographic information of focus group and interview participants.

Characteristics	6-mo interview and focus group participants only		6- and 12-mo interview and focus group participants		12-mo interview and focus group participants only	
	GPs^a^ (n=41)^b^	Practice managers (n=6)	GPs (n=8)	Practice managers (n=1)	GPs (n=1)	Pediatricians (n=2)
Gender, n (%)^c^						
Female	27 (66)	—^d^	6 (75)	—	1 (100)	1 (100)
Male	12 (29)	—	2 (25)	—	0 (0)	1 (100)
Prefer not to say	2 (5)	—	0 (0)	—	0 (0)	0 (0)
Modified Monash Model classification, n (%)						
MM1^e^	18 (44)	2 (33)	5 (62)	0 (0)	0 (0)	2 (100)
MM3^f^	11 (27)	1 (17)	0 (0)	0 (0)	0 (0)	0 (0)
MM4^g^	2 (5)	1 (17)	2 (25)	1 (100)	1 (100)	0 (0)
MM5^h^	10 (24)	2 (33)	1 (13)	0 (0)	0 (0)	0 (0)
Pediatric patients seen per week (GPs only), n (%)						
<11	17 (41)	—	1 (13)	—	0 (0)	—
11-20	17 (41)	—	5 (62)	—	1 (100)	—
>20	7 (17)	—	2 (25)	—	0 (0)	—
Years of practice, n (%)						
<6	15 (37)	—	3 (37)	—	0 (0)	0 (0)
6-15	16 (39)	—	3 (37)	—	1 (100)	1 (50)
>15	10 (24)	—	2 (25)	—	0 (0)	1 (50)
Formal pediatric training (GPs only)^i^, n (%)						
Yes	14 (34)	—	4 (50)	—	1 (100)	—
General practice type, n (%)						
Solo GP	1 (2)	—	1 (13)	—	0 (0)	—
Group practice	40 (98)	—	7 (87)	—	1 (100)	—
Billing type (for patients aged <18 y), n (%)						
Bulk-billing only^j^	20 (49)	—	3 (37)	—	1 (100)	—
Private billing only^k^	9 (22)	—	1 (13)	—	0 (0)	—
Mixed billing^l^	12 (29)	—	4 (50)	—	0 (0)	—

a GP: general practitioner.

b Demographic information from 3 GPs who participated in 6-month focus groups was not available.

c Gender of practice managers was not formally assessed.

d Not applicable.

e MM1: metropolitan areas.

f MM3: large rural towns.

g MM4: medium rural towns.

h MM5: small rural towns.

i Completion of pediatric training outside of MBBS, MD, or GP Registrar training, including Diploma of Child Health, Sydney Child Health Program prior to SUSTAIN.

j Bulk-billing: GPs accept Medicare (Australia’s publicly funded universal health insurance scheme) benefit as full payment for service, no charge to patient.

k Private billing: patient pays for all or part (“gap”) of medical services, Medicare rebate can be claimed by patient for some consultations afterward. and Medicare rebate can be claimed by patient for some consultations afterward.

l Mixed billing: GP bulk-bills some patients, that is, those on health care card or other concession, and others are privately billed.

### Theme 1: Compatibility and Adaptability of the SUSTAIN Model as Drivers of Seamless Delivery and Engagement

SUSTAIN was positively received by participants, both the overall model of care and the individual components. As outlined in Textbox 1, participants consistently described the model as well organized and feasible within routine general practice workflows.

*I do think it’s a brilliant idea. It’s an absolutely brilliant idea, and it’s a pity that we can’t do this across all the specialties*

[GP26, month 6, MM4]

GPs and practice managers reported a clear understanding of the model of care and described the onboarding process as straightforward, with sufficient resources to integrate SUSTAIN into routine practice with minimal disruption. GPs and pediatricians highlighted variability in how the model was used across practices and individuals, noting that its flexibility was highly valued. Consistent with the CFIR innovation and inner setting domains, Textbox 1 presents that GPs viewed the SUSTAIN model as highly compatible with general practice workflows and its adaptability was seen as a key strength that allowed it to meet the needs of GPs. GPs reported engaging with components of the model that best suited their clinical style, experience, confidence, and workload, with some preferring phone or email support over coconsultations due to scheduling constraints. These reported preferences further illustrate how adaptability supported engagement of individual GPs. Pediatricians also reflected that some GPs would only engage with one component of the model, and this appeared to provide them with the level of support that was required:

*There were some GPs who clearly just had Thursday one o’clock in their diaries...and I’ve seen them pop in, and I go, “what case you want to talk about?” “Oh, yeah, there is a child I want to talk about that I saw three days ago, what about this? What about that?” Because that worked for them and their workflow.*

[P2, end of intervention, MM1]

Coconsultations were typically reserved for more complex cases, clinical uncertainty, or when parents sought additional reassurance from a pediatrician. Phone or email support and “lunch and learn” sessions were reported as being predominantly used for general queries or recent cases that did not warrant coconsultation. GPs who engaged with the SCHP described it as informative and comprehensive, using it either systematically or to address specific knowledge gaps. However, many GPs reported that they did not participate in the SCHP due to time constraints. Several GPs reported that they preferred the informal and flexible “lunch and learn” sessions, which allowed them to join as needed and better suited the demands and unpredictability of general practice. Together, these findings demonstrate that perceived compatibility with workflow and adaptability to clinician needs were central drivers of GP engagement with the SUSTAIN model, as detailed in Textbox 1.

Textbox 1. **Consolidated Framework for Implementation Research (CFIR) constructs and participant quotations for theme 1.**

Compatibility and adaptability of the SUSTAIN model as drivers of seamless delivery and engagement

**CFIR constructs**

- Innovation and inner setting domains: SUSTAIN is compatible with general practice workflow, systems, and processes and adaptable to the needs of the general practitioners (GPs) and general practice clinics.
- Individuals domain: SUSTAIN addresses the need of GPs in pediatrics (increased support, confidence, and knowledge building)

**Illustrative quotes**

- Model of care

-  “The communication was really good. The emails were great…from a running perspective I just think that it was organized well” (GP14, month 6, MM1).
-  “I think it's great. Yes, the ultimate solution to paediatric care is to get more paediatricians available, but I'm aware that this project is not aiming to do that, in terms of what this model aims to do, I love it” (GP11, month 6, MM4).

- Coconsultations

-  “I've seen a couple of co-consults, which were great. [SUSTAIN paediatrician]’s been really approachable, really helpful, emailing me, happy for me to ring in, easy to make an appointment. All of that's fantastic” (GP27, month 6, MM4).

- Phone or email support

-  “I found it [email support] very useful because I got two very, very prompt and very, very appropriate answers” (GP11, end of intervention, MM4).

- “Lunch and Learn”

-  “They [‘Lunch and Learn’] have been great. I wish I could make it to more of them. But I do try to make it to as many as I can. And they're really valuable. It's that practical application of knowledge, which you'd never find in an online resource or a textbook, anywhere” (GP28, month 6, MM3).

- Sydney Children’s Health Program (SCHP)

-  “It [SCHP] was really comprehensive. I didn't expect it to have such a broad range of topics and a lot of stuff that I hadn't come across since medical school, just to refresh my memory, learn a bit more with regards to areas that I hadn't had any experience in, and just feeling a little bit more prepared if something does come up left field in clinic” (GP30, end of intervention, MM4).

### Theme 2: The SUSTAIN Model’s Relative Advantage in Reinforcing GPs’ Pediatric Care Scope, Access, and Motivation

#### Overview

As summarized in Textbox 2, theme 2 predominantly aligned with the CFIR construct of innovation relative advantage within the innovation domain and mapped to key constructs within the inner setting and individuals domains. Participants identified increased efficiency and working at the top of the scope for GPs as the major benefits arising from participation in the SUSTAIN model.

Textbox 2. **Consolidated Framework for Implementation Research (CFIR) constructs and participant quotations for theme 2.**

The SUSTAIN model’s relative advantage in reinforcing general practitioners’ (GPs) pediatric care scope, access, and motivation

**Efficiency**

- CFIR construct

-  Innovation domain: The SUSTAIN model offers a relative advantage over normal practice.

- Illustrative quotes

-  “It's been really good. It's quite handy being able to access a paediatrician when we want to and being able to speak directly with the consultant as opposed to going to switchboard, and then waiting for switch to call the registrar, and then the registrar may not be certain on their advice and running it by the consultant before getting it back to us. So it's much more useful” (GP20, month 6, MM1).
-  “[SUSTAIN paediatricians] reply very quickly, and I get good advice in a timely manner that I can actually use it and utilize it for the patients” (GP49, month 6, MM1).
-  “There would have been a few that have saved a referral because I could email and ask” (GP1, month 6, MM3).

**Working at top of scope**

- CFIR construct

-  Inner setting domain: GPs and pediatricians have shared values and beliefs to care, prioritizing recipient-centered care and supporting and addressing the needs of the children and families.
-  Individuals domain: Model addressed the need of GPs (increased support, confidence, upskilling, and knowledge building in pediatrics) and the need of families (reassurance and accessible pediatric care).

- Illustrative quotes

-  “I think I referred a lot, anyway, but it's definitely saved me some more referrals than I would have done” (GP33, month 6, MM1).
-  “One of the things I really appreciate is sort of you pick up some tips, even just by observation, how a person takes a history, how they react to maybe a child who doesn't want to talk. I don't know if I said this to [SUSTAIN paediatrician], but I really liked their low key, calm response and you take that on board. So it's like I say there's a bit of art to medicine, you pick up a bit of the art also” (GP44, month 6, MM3).

#### Increased Efficiency

Consistent with the CFIR construct of innovation relative advantage (Textbox 2), participants described SUSTAIN as a more streamlined alternative to standard referral procedures and pathways, which often involved contacting hospital switchboards, speaking with rotating registrars, and waiting extended periods for feedback:

*To have that consistency to talk to someone as opposed to just calling the hospital and speaking to a new reg every time who doesn’t know you, and you’re interrupting their day. I’ve had some really complex cases that [SUSTAIN paediatrician] has kind of really guided me on or just confirmed what I was doing. I think it’s excellent, I would love it to continue.*

[GP18, month 6, MM5]

Access to phone or email support, coconsultations, and “lunch and learn” sessions enabled timely advice, allowing GPs to provide prompt feedback to families. Direct specialist-to-specialist communication with a consultant pediatrician was reported as being particularly valuable in reducing delays and administrative burden on GPs and general practice clinics:

*We don’t even have to write a referral, we just write “this is my question” and then get to have that two-way conversation in the actual co-consult itself, and then and you get the immediate feedback and not needing to wait any period of time. And I’m imagining that saves a fair bit of time writing things out and typing and sending from the administrative side of things from the specialist end as well.*

[GP28, month 6, MM3]

In some cases, GPs reported that this immediacy of advice reduced or avoided referrals altogether, while in others it streamlined escalation to subspecialty care. These findings reinforce the perceived efficiency gains and comparative advantage of the model over usual care.

#### Working at Top of Scope

Theme 2 also aligned with the CFIR construct related to GP-pediatrician shared values focused on recipient-centered care, as well as the SUSTAIN model’s responsiveness to GP needs for additional support. Most GPs indicated that their capacity to provide care, support, and management of pediatric conditions had improved through participation in the SUSTAIN model:

*Each issue I’m managing is making me more confident that the next time I can do this without [SUSTAIN paediatricians] because now I know what to do.*

[GP49, month 6, MM1]

GPs reported increased confidence to manage conditions they may previously have referred, including mental health concerns, constipation, enuresis, and failure to thrive. Several also described improved judgments regarding when escalation to acute or subspecialty services was appropriate. Even highly experienced GPs reported gaining new insights, practical techniques, and resources that strengthened their practice:

*[SUSTAIN] would save me referring probably 5 to 10 people a year.*

[GP48, month 6, MM1]

For GPs who saw fewer children, SUSTAIN was reported to provide accessible support and education around best practice guidelines and referral options. GPs who attended the “lunch and learn” sessions indicated that the learning was 2-fold, with discussions with the SUSTAIN pediatricians and other GPs facilitating ideas, insights, and knowledge building. As detailed in Textbox 2, the perceived relative advantage of SUSTAIN, combined with its impact on GP confidence and capability, underpinned its value in supporting clinicians to work at the top of their scope as GPs.

### Theme 3: GP and Pediatrician Relational Connections and Communication Strengthening Collaboration and Trust

#### Overview

The critical role that professional relationships have in strengthening collaboration and building trust was highlighted by participants. As outlined in Textbox 3, theme 3 mapped primarily to constructs within the inner setting domain, particularly relational connections, communication practices, and shared culture and values centered on recipient-focused care.

Textbox 3. **Consolidated Framework for Implementation Research (CFIR) constructs and participant quotations for theme 3.**

General practitioner (GP) and pediatrician relational connections and communication strengthening collaboration and trust

**Collaboration**

- CFIR construct

-  Inner setting domain: SUSTAIN GPs-pediatrician relational connection, communication and shared culture, values and beliefs to care, and prioritizing recipient-centered care.

- Illustrative quotes

-  “They've [SUSTAIN paediatricians] got an understanding of rural, not everybody does” (GP28, month 6, MM3).
-  “I was surprised by some of the complexity that they [GPs] were having to manage, especially the rural and remote GPs...and managing stuff with very limited resources” (P1, end of intervention, MM1).
-  “I think once you get to know the paediatrician you are asking the advice from, it just makes it much easier...it's just so much easier to get advice on kids than it was before SUSTAIN” (GP50, month 6, MM1).
-  “I think the concept is just so good, it’s such a good idea. Especially if you have a relationship with the specialist. So I've met [SUSTAIN paediatrician] in the lunchtime meetings and so it's a lot easier for me to present a case to someone that I've already met, even though I'm only meeting through an audio visual, I think it works a lot better” (GP26, month 6, MM4).

**Strengthening family trust in GP-delivered care**

- CFIR construct

-  Inner setting domain: The GP and pediatrician hold shared values, beliefs, and norms around caring, supporting, and addressing the needs of children and families.
-  Individuals domain: Model addressed the need of families to be heard. Model helps build family trust in GP capability.

- Illustrative quotes

-  “I think the parents really find the co-consults valuable in terms of they really feel like they got something out of it” (GP2, month 6, MM1).
-  “It actually, I think, strengthened the relationship for the GP and the family, because I could go, ‘actually, no, everything's sweet, you've done more than what I would have done, so it's great, you've actually covered everything off, and you don't need the specialist’” (P2, end of intervention, MM1).
-  “I think once they've heard that once from the paediatrician, they're more likely to then take on board what we're saying down the track, just because they've had those two different sources telling them the same thing, and it's ended up okay. So I think it also helps with the relationship with the families and us as the GPs as well” (GP30, end of intervention, MM4).

#### Collaboration Between GPs and Pediatricians

Collaboration between practitioners emerged as a key factor in the successful implementation of the SUSTAIN model. Participants described how opportunities for sustained, ongoing interaction fostered mutual understanding of professional contexts, including the complexity of cases managed in general practice and the time pressures faced by GPs, particularly in rural settings. These findings reflect strong relational ties and shared values within the inner setting domain (Textbox 3):

*Just generally, I don’t think you could find better people than [SUSTAIN paediatricians] to kind of be that great resource, technically very astute, very capable, but then also be that kind of person that GPs just feel really comfortable talking to, because not everyone is. And if I was to think about the program in the future, or any program in the future, lots of it is about assessing for fit, so that the clinicians you put in it are actually good at talking to GPs, because not everyone is.*

[GP41, end of intervention, MM1]

GPs emphasized the value of specialist-to-specialist partnerships, describing rich learning opportunities supported by safe and inclusive learning environments. The multiple points of contact, including coconsultations and “lunch and learn” sessions, strengthened continuity and deepened professional relationships over time:

*I suppose just no question felt silly...every question was taken on board, they made you feel like it was interesting, it was helpful information. I think that was really useful.*

[GP2, month 6, MM1]

Virtual delivery via telehealth was not viewed as a barrier to relationship development, reflecting adaptation to postpandemic models of care. Although some GPs suggested that optional face-to-face interaction may enhance connection, telehealth was generally considered acceptable and effective, particularly in rural contexts.

#### Strengthening Family Trust in GP-Delivered Care

Theme 3 also aligned with CFIR constructs within the inner setting domain related to shared values and beliefs supporting recipient-centered care and to constructs within the individuals domain, particularly around addressing family needs and reinforcing trust (Textbox 3).

GPs reported that having the support of the SUSTAIN pediatricians, whether through coconsultation or phone or email support, helped reassure families that their child’s medical problem was being appropriately managed by the GP. Participants reported that this reassurance helped increase family confidence in GP-led management and supported continuity of care within primary practice:

*I’m finding that families are more willing to keep following up with us, as opposed to just waiting for the specialist or going into hospital if things get dire.*

[GP30, month 6, MM4]

In some cases, collaborative input changed the trajectory of a patient’s journey, reducing the need for referral; in others, it enabled more GPs to feel more confident to manage the “work up” required for specialist care. GPs reflected that this streamlined approach saved time and reduced the burden for families, further reinforcing trust in GP-delivered care. As detailed in (Textbox 3), relational connection and shared commitment to child- and family-centered care were central to strengthening both interprofessional collaboration and family confidence in GP-delivered care.

### Theme 4: Relative Priorities, Needs, and Available Resources as Key Moderators of Participants’ Engagement With the Model

Heavy clinical workload and limited time were consistently identified as key barriers to engagement with the SUSTAIN model. Consistent with the relative priority construct within the CFIR inner setting domain (Textbox 4), many GPs described SUSTAIN as valuable yet challenging to prioritize within constraints of busy practice environments. GPs seeing fewer pediatric patients (less than 11 per week) reported limited engagement across the intervention period, reflecting a lower perceived need for support. Coconsultations and “lunch and learn” sessions were reported as being particularly affected by time pressures. Scheduling coconsultations was described as challenging in the context of heavily booked clinical calendars, especially in smaller or solo practices. Many GPs commented on the difficulties they faced getting to a “lunch and learn” session due to clinical overrun, administration requirements, and the need for rest between sessions, while evening sessions were often reported as not being feasible due to personal commitments. These findings reflect the influence of work infrastructure and resource constraints within the Inner Setting (Textbox 4).

*I think the big thing is, we are so under the pump, our wait times are six weeks to book an appointment...there’s no way I can easily fit that [co-consultation] in my schedule at the moment to actually work. So it’s just not practical. The idea is good but I just don’t think it’s practical.*

[GP 6, month 6, MM5]

Variation in engagement also reflected differences in perceived need, consistent with the CFIR individuals domain (Textbox 4). While many experienced GPs acknowledged that SUSTAIN offered skills and knowledge development, others felt confident managing complex pediatric presentations independently and did not perceive a strong need to engage. Some GPs practicing in rural settings described having adapted over time to managing complexity without specialist input:

*I’ve been here for a long time, I’ve been seeing kids for a long time, I’ve already got a pattern on what I do for kids. I’ve got a pattern of where I refer and why I refer and there isn’t a local paediatric service... and so SUSTAIN, I hadn’t really felt a perceived need for a new system, so I haven’t engaged with SUSTAIN very much, that’s my perspective on it.*

[GP5, month 6, MM5]

In addition, family expectations and preferences influenced use of the SUSTAIN model. Several GPs reported that parents often requested direct referral to a pediatrician in the community and could be insistent on this course of action over engagement in the SUSTAIN model. Together, these findings demonstrate that engagement with SUSTAIN was moderated by competing priorities, structural constraints, and perceived need for pediatric support, as detailed in Textbox 4. While the model was broadly valued, its uptake depended on the alignment between GP and practice capacity, clinician motivation, and perceived need and family expectations.

Textbox 4. **Consolidated Framework for Implementation Research (CFIR) constructs and participant quotations for theme 4.**

Relative priorities, needs, and available resources as key moderators of participants’ engagement with the model.

**Workload, time available, and needs of general practitioners (GPs) and families**

- CFIR constructs

-  Inner setting domain: The implementation of SUSTAIN can be impacted by its relative priority within general practices, as well as the work infrastructure and available resources of these settings.
-  Individuals’ subdomain: The perceived need for pediatric support through SUSTAIN.

- Illustrative quotes

-  “The co-consultations and the ‘Lunch and Learn’ sessions and the email, it's all really helpful. It's just, I guess the nature of GP isn't an environment that's conducive to ongoing learning really” (GP1, month 6, MM3).
-  “After so many years practicing in an environment, I mean, look, we're deprived of many specialties on tap, so you adjust and you become more comfortable with managing more complex things, can't help it, I mean, you sink or swim” (GP37, month 6, MM3).
-  “There wasn't always a lot in there [‘Lunch and Learn’ sessions] for those of us who've been doing this for a little while” (GP38, month 6, MM3).
-  “A lot of times the mums just really want to see a paediatrician. So typically, I try to avoid referring if I don't think it's necessary, but sometimes they're quite persistent” (GP46, month 6, MM5).

### Theme 5: Funding and Enhancement of Model Design: Considerations for Sustainability

Participants emphasized that long-term sustainability would depend on refinements to both model design and funding structures to support the model’s implementation.

#### Model of Care Design

Most GPs commented favorably on the design of the SUSTAIN model; however, they also identified limitations that reduced its perceived relevance in certain clinical contexts. In particular, the defined boundary that the SUSTAIN pediatricians would not assume ongoing clinical responsibility for children’s care limited the model’s perceived applicability and relevance for some GPs. This was noted particularly in relation to the diagnosis and management of children with autism spectrum disorder and/or attention-deficit hyperactivity disorder. Mapping to the CFIR implementation process domain, participants indicated a potential need for adapting the model to better reflect and accommodate clinical realities (Textbox 5).

*The reason I haven’t used it much....90% of what I send off to the paeds is behavioural. They are people who are wanting a diagnosis and wanting medication and you guys can’t do that. So that’s been my main reason for not using you more.*

[GP27, month 6, MM4]

The SUSTAIN pediatricians similarly reflected that flexibility in how the model is operationalized would be important for long-term implementation with suggestions of increased scope for the pediatrician to take more clinical responsibility, if clear professional and clinical boundaries were in place. Conversely, several GPs proposed that certain components, such as the frequency of “lunch and learn” sessions or pediatrician availability, could be reduced while maintaining value, potentially supporting widespread roll-out across the state and broader scale-up. A hybrid model incorporating periodic face-to-face engagement alongside telehealth was also suggested by GPs and the SUSTAIN pediatricians as a means of strengthening connection and contextual understanding (Textbox 5).

Textbox 5. **Consolidated Framework for Implementation Research (CFIR) constructs and participant quotations for theme 5.**

Funding and enhancement of model design: considerations for sustainability

**Model of care design**

- CFIR constructs

-  Implementation process domain: adapting the design of the sustain model to meet gp and general practice needs.

- Illustrative quotes

-  “An ideal scenario for us in terms of [a] more focused and intense model than SUSTAIN, so that combination of telehealth, phone a friend, but then also having that paediatric team, or someone on the team come out and spend a couple of days consulting every two months...to me, the combination of telehealth/face to face would be perfect” (GP11, month 6, MM4).
-  “It probably would be easier if they came and visited face to face. And you know, every 6 months you had a paediatrician, and you just booked a whole day of kids and had the paediatrician sit with you for a whole day like you would learn a lot that way as well” (GP1, month 6, MM3).
-  “While virtual is great and makes it accessible for people, if this was a long term roll out permanent type role, I'd be saying what the paediatrician actually needs is to go out to that area that's regularly using the service, meet the GPs, meet the paediatric service, meet the paediatric service that doesn't exist, whatever it might be to actually get a feel for what they're working in” (P2, month 12, MM1).

**Financial considerations**

- CFIR constructs

-  Outer setting domain: The availability of funding to implement the SUSTAIN model of care in an ongoing capacity.

- Illustrative quotes

-  “If it's going to be long term, then of course the paediatricians have to be remunerated for their time and if we're going to be doing longer consults, then perhaps we have to have like an additional thing [financial incentive] attached to it” (GP10, month 6, MM1).
-  “For a while there was a Medicare item number where we could do a co-consultation, but I think that was stopped, and I found that a useful thing. I think we've got to move towards this because we're never going to solve the problem of enough specialists, especially in rural areas” (GP26, month 6, MM4).

#### Financial Considerations

Sustainability was also strongly linked to the availability of funding structures within the Outer setting domain. Although financial barriers did not prevent participation during the trial, many GPs indicated that long-term engagement would depend on appropriate remuneration. Participants highlighted that current Medicare billing arrangements do not adequately support coconsultation models or longer appointments involving 2 clinicians. GPs, practice managers, and pediatricians agreed that either revised billing items or supplementary financial incentives would be required to support routine implementation. Without structural funding alignment, participants expressed concern that the model’s scalability and sustainability would be limited.

Overall, as detailed in Textbox 5, sustained implementation of SUSTAIN would require adaptive refinement of the model design and modification of funding mechanisms to ensure feasibility within primary care.

## Discussion

### Principal Findings

GPs, practice managers, and SUSTAIN pediatricians found the model acceptable, highlighting its flexibility and practicality. GPs valued the learning, collaboration, and specialist support, with the virtual format viewed as an effective way to access non-GP specialist input that would otherwise be unavailable to many general practice clinics. Key benefits included improved workflow efficiency, capacity building in pediatric care, enhanced professional relationships, and increased family trust in GP-delivered care. However, time constraints and workflow pressures within general practice limited engagement, reflecting broader international concerns about primary care sustainability [32-34]. Participants also emphasized that appropriate Medicare reform or alternative remuneration to support coconsultation would be essential for the long-term sustainability of the model.

The findings that the SUSTAIN model of care was acceptable to GPs, practice managers, and pediatricians align with results from the SC4C trial and the Children and Young People’s Health Partnership model, further supporting the value of integrated GP-pediatrician models in general practice [35,36]. Its acceptability in rural general practice clinics demonstrates utility beyond metropolitan settings, consistent with NSW Health priorities [37] and extends previous research by examining an entirely virtual model of care across both metropolitan and rural contexts. In line with the UK Medical Research Council guidance on complex interventions, which emphasizes responsiveness to the implementation context [38], uptake of SUSTAIN components was shaped by practice capacity, resources, and GP learning preferences, with participants highlighting that the model’s flexible design was better suited to primary care than a prescriptive “one-size-fits-all” approach.

GPs reported that the SUSTAIN model of care provided an efficient and effective pathway to non-GP specialist pediatric support, providing timelier and streamlined access for both GPs and families compared to usual practice. These findings align with other primary care–specialist integration models in Australia [39-42] and internationally [43-45], where improved workflow and management of pediatric patients were similarly observed. Such efficiencies are particularly important in the context of workforce shortages, long working hours, and high burnout among GPs, particularly in rural areas, and may contribute to improved flexibility, work-life balance, and career longevity for GPs [32,33,46-48]. However, the absence of dedicated Medicare items for coconsultation or additional financial incentives provided to general practice clinics to support GP specialist collaboration was identified as a potential barrier to long-term sustainability. This concern reflects findings from the 2024 Royal Australian College of General Practitioners Health of the Nation survey, in which more than half of GPs reported income maintenance as a challenge [46]. Participating GPs who bulk-bill children (aged <16 y) noted financial losses associated with longer consultations compared with shorter standard appointments, including in the context of triple pediatric (aged <16 y) bulk-billing incentives introduced in Australia in 2023 [49]. Attendance at “lunch and learn” sessions and engaging in phone or email advice were also typically reported as being unpaid. Scaling up of models of multidisciplinary integrated care and supporting GPs to work at the top of scope have been recognized as key objectives of primary health care into the future at both national and state levels in Australia [37,50,51]. Providing the financial mechanisms to facilitate these policy objectives and support models such as SUSTAIN to be implemented ongoing is vital.

Despite growth in Australia’s pediatric population and increasing rates of childhood chronic illness, GP consultations with children have declined in number and duration [52]. While this trend has been attributed to greater demand from adults and older patients, concerns have been raised that reduced exposure may diminish GP confidence in managing chronic disease and behavioral concerns in children [52]. In contrast, GPs participating in SUSTAIN reported increased confidence and capacity to manage pediatric presentations through education, observation, and improved access to specialist advice and referral pathways, with perceived benefits for patient outcomes. These findings are consistent with the SC4C study, where GPs similarly reported enhanced confidence, knowledge, and skills in pediatric care [35] and reflect similar findings from international studies [43-45,53], supporting the potential of integrated GP-pediatrician models strengthen primary care management of children’s health concerns.

Concurrently, the burden of disease and subsequent landscape of pediatric general practice in Australia, and internationally, has shifted toward more complex neurodevelopmental, behavioral, and mental health presentations [54-60], increasing demand for accessible assessment and management pathways particularly for conditions such as attention-deficit hyperactivity disorder [54,61,62]. Evidence suggests that GPs are willing to take a greater role in managing developmental and behavioral conditions when appropriately supported [63,64] aligning with NSW Health recommendations to enhance GP training and support in this area [65] and recent policy reforms expanding prescribing rights for GPs to care for pediatric patients requiring stimulants in NSW [66,67]. Within SUSTAIN, GPs reported improved confidence in undertaking developmental and behavioral assessments, including preparatory work prior to referral; however, some indicated that further adaptation of the model, such as incorporating structured shared care or temporary pediatrician-led management, would be required to fully support complex neurodevelopmental care in primary practice.

International research examining the implementation of integrated pediatric health models has consistently identified the development of a trusted professional relationship between practitioners as fundamental to success [68]. In this study, continuity of interaction through SUSTAIN enabled pediatricians and GPs to build meaningful relationships and mutual trust, reflecting findings from the SC4C trial that emphasized the importance of open, nonjudgmental communication, clear role delineation, and psychologically safe collaborative environments in effective integrated care delivery [35]. These findings are reinforced by a systematic review highlighting that clearly defined roles, shared understanding of collaborative benefits, and dedicated time for communication are key to developing successful interprofessional teamwork in primary care [69], alongside further research demonstrating that regular communication and explicit expectations underpin trust between GPs and non-GP specialists [70-72]. Unlike many comparable pediatric integrated care models that incorporate face-to-face interaction [6-9], SUSTAIN was delivered entirely virtually; however, participants did not perceive the remote format as a barrier to relationship development. Although research specifically examining virtual GP-pediatrician collaboration remains limited, studies of telehealth coconsultation between tertiary specialists and rural clinicians suggest positive experiences and reduced professional isolation [73,74], while evidence from virtual communities of practice indicates that online engagement can mitigate hierarchical barriers and foster equitable, accessible knowledge exchange for rural practitioners [75].

GPs and pediatricians perceived that participation in SUSTAIN strengthened family trust in GP-delivered care, increasing families’ willingness to continue engaging with their GP for future health concerns, consistent with findings from the SC4C trial where families reported greater confidence in their GP’s pediatric care following participation [35]. These outcomes are particularly relevant, given current help-seeking patterns, with children older than 15 years accounting for the highest rates of lower urgency (ED) presentations and 13% of potentially preventable hospitalizations in Australia [76], and 39% to 60% of pediatric ED visits classified as nonurgent across several Organisation for Economic Co-operation and Development countries [77-81]. Dissatisfaction or mistrust of primary care has been identified as a contributor to nonurgent ED use [82-84], with 1 Australian study reporting that only 45% of parents felt completely confident in GP-delivered care and nearly half preferred pediatrician-led care for their child [83]. Although evidence on telehealth-delivered integrated care models for children remains limited, prior research suggests telehealth can enhance family perceptions of primary care providers [73]; this study extends that literature by highlighting coconsultation and timely pediatric input within primary care may strengthen family confidence and trust, particularly in rural contexts where telehealth may improve access to care.

### Strengths and Limitations

This qualitative analysis had broad engagement from GPs and practice managers across metropolitan and rural general practice clinics within NSW. This representation is a strength and provides evidence for the utility of the SUSTAIN model across a range of settings. However, participating practices were self-selecting. Despite attempts to actively recruit a diverse range of practices and to reach GP practices that care for underrepresented or harder-to-reach populations groups, for example, remote, regional, rural general practices, the cohort of participating practices may be more motivated to participate in research and seek opportunities for professional development. This potentially limits the generalizability of results. For example, although practices participating in the SUSTAIN model were in small, medium, and large rural towns (MM3 to MM5), there were no practices located in remote (MM6) or very remote (MM7) communities in NSW. Further studies to account for additional factors impacting uptake of the model in these communities are required.

As outlined in the *Methods* section, GPs who had not engaged with any component of the model were invited to participate in the interviews and focus groups. However, only one consented to take part. As a result, the findings of this study may not fully capture the perspectives of all the GPs exposed to the model, particularly those with very limited engagement. Furthermore, there was heterogeneity in how GPs engaged with the SUSTAIN model, with individual engagement reflecting differences in preference, clinical style, and workload. As a result, the qualitative themes within this study reflect participants’ subjective experiences of the SUSTAIN model as they encountered it. Although variability in exposure to different components is an inherent feature of flexible, practice-embedded implementation models such as SUSTAIN, we acknowledge this as a limitation in the generalization of results.

This study only included perspectives from GPs, practice managers, and the SUSTAIN pediatricians as we were unable to successfully recruit additional practice staff (practice nurses and administrative personnel) to participate. Understanding the implementation of the SUSTAIN model of care from the perspective of the broader practice team is an important consideration for future implementation.

### Conclusions

The SUSTAIN model of care expands on our understanding of the benefits of integrated GP-pediatrician models of care in general practice by demonstrating the utility of a pediatrician supporting a GP in their practice via telehealth across metropolitan and rural environments. The nature of the model, with virtual support from the pediatrician, has the potential to improve access to quality health care for children by reducing barriers that many rural and remote families currently face in accessing and affording health care close to home. However, these findings should be interpreted in light of study limitations, including participant self-selection, heterogeneity in engagement with the model, and the absence of remote and very remote (MM6 and MM7) general practice clinics, which may limit generalizability. For the SUSTAIN model to be sustainable and scalable, there is a need for a flexible design to suit the requirements of different general practice clinics and consideration of remuneration to adequately compensate clinicians working within a coconsultation model of care.

## Supplementary material

10.2196/86707 Multimedia Appendix 1 SUSTAIN_A virtual integrated GP-pediatrician model of care.

10.2196/86707 Checklist 1 SUSTAIN_A virtual integrated GP-pediatrician model of care_COREQ checklist.

## Abbreviations

CFIR: Consolidated Framework for Implementation Research
COREQ: Consolidated Criteria for Reporting Qualitative Research
ED: emergency department
EOI: expression of interest
GP: general practitioner
MM: Modified Monash (classification)
NSW: New South Wales
REDCap: Research Electronic Data Capture
SC4C: Strengthening Care for Children
SCHP: Sydney Child Health Program
UNSW: University of New South Wales
