# Supplementary material for: A Virtual Integrated General Practitioner–Pediatrician Model of Care Implemented in Metropolitan and Rural Primary Care Settings: Qualitative Analysis of Clinician Perspectives on the SUSTAIN Model of Care
Source: J Med Internet Res. 2026 May 5;28:e86707. doi: 10.2196/86707 (PMC13143162; doi:10.2196/86707)
Supplement: Multimedia Appendix 1 [file jmir-v28-e86707-s001.docx]

**Supplemental Figure 1. Overview of number of GP engagements in co-consultations, phone/email support and ‘Lunch and Learn’ sessions.**

**
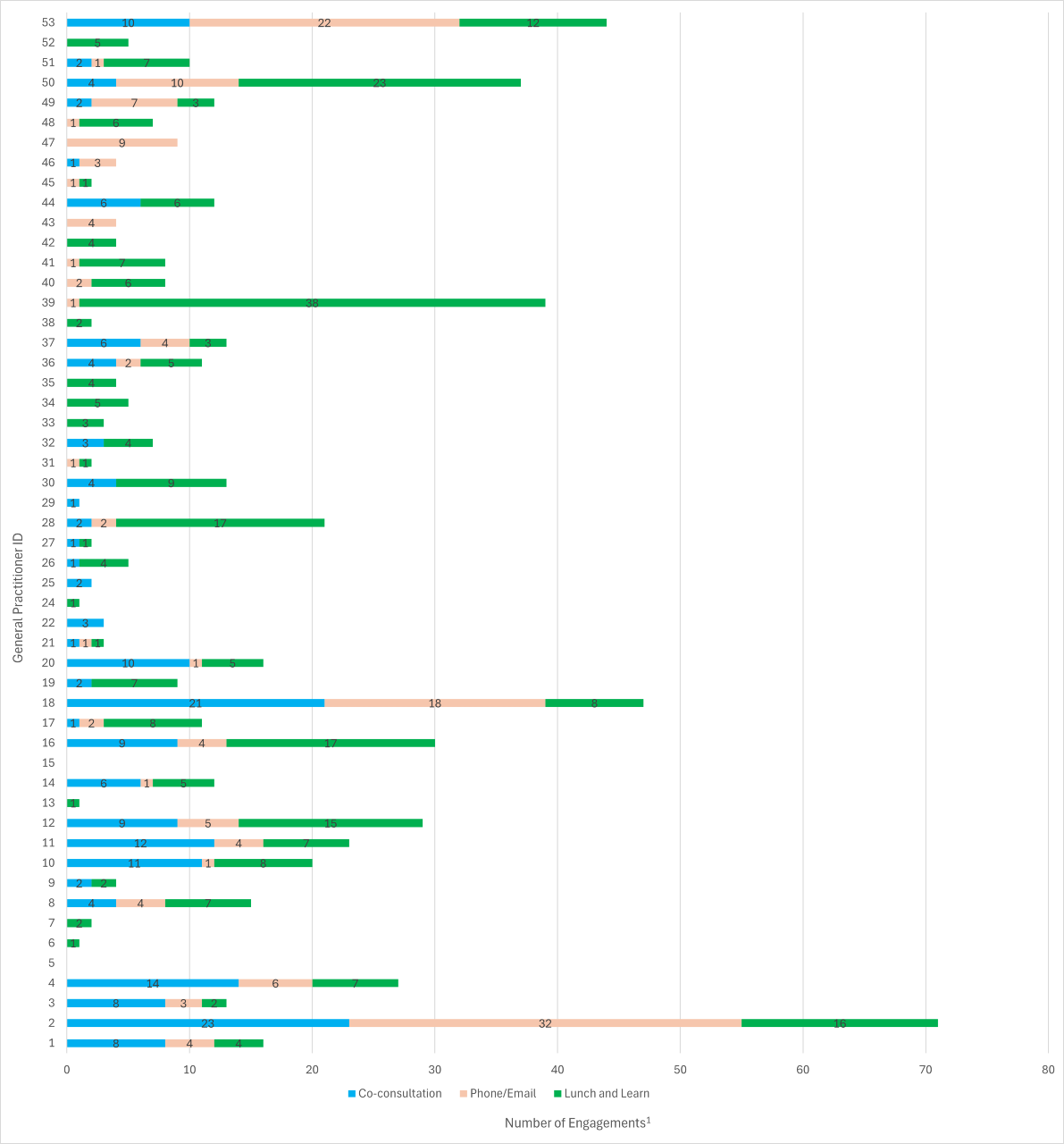
**

*^1.^ One engagement was equivalent to GP attendance at one co-consultation, one phone-call, text message or email support sent by GP to SUSTAIN paediatrician, one attendance at ‘Lunch and Learn’ session.*

**Supplemental Table 1. Overview of GP participant engagement in Sydney Child Health Program by time (hh:mm:ss)**

| **General Practitioner ID** | **Time spent on program (hh:mm:ss)**^1^ |
| --- | --- |
| 1 | 0:43:27 |
| 2 | 7:33:52 |
| 3 | 0 |
| 4 | 2:43:27 |
| 5 | 0 |
| 6 | 0 |
| 7 | 0 |
| 8 | 2:35:05 |
| 9 | 74:54:07 |
| 10 | 28:53:12 |
| 11 | 0:58:32 |
| 12 | 11:00:24 |
| 13 | 0 |
| 14 | 0 |
| 15 | 0:00:32 |
| 16 | 5:48:11 |
| 17 | 0 |
| 18 | 0 |
| 19 | 8:24:53 |
| 20 | 0:42:39 |
| 21 | 0 |
| 22 | 0 |
| 24 | 0 |
| 25 | 0 |
| 26 | 52:40:33 |
| 27 | 0 |
| 28 | 0:04:06 |
| 29 | 0:46:21 |
| 30 | 133:41:35 |
| 31 | 0 |
| 32 | 0 |
| 33 | 0 |
| 34 | 0 |
| 35 | 39:25:44 |
| 36 | 1:18:37 |
| 37 | 0 |
| 38 | 0 |
| 39 | 299:24:27 |
| 40 | 0:23:24 |
| 41 | 0 |
| 42 | 0 |
| 43 | 0 |
| 44 | 1:55:24 |
| 45 | 22:13:25 |
| 46 | 0:03:57 |
| 47 | 0 |
| 48 | 0 |
| 49 | 0:02:38 |
| 50 | 1:05:27 |
| 51 | 0:00:01 |
| 52 | 0:00:03 |
| 53 | 129:22:38 |

*^1.^ A genuine engagement with the SCHP was considered if a participant spent more than 1 minute on learning within the program*
